# Supplementary material for: Cold Atmospheric Plasma Decontaminates Arabidopsis thaliana Seeds and Remodels Seedling Fungal Microbiota
Source: Plants (Basel). 2026 Sep 4;15(17):2719. doi: 10.3390/plants15172719 (PMC13568275; doi:10.3390/plants15172719)
Supplement: Supplementary file 1 [file plants-15-02719-s001.zip › Supplemental Figs.pdf]

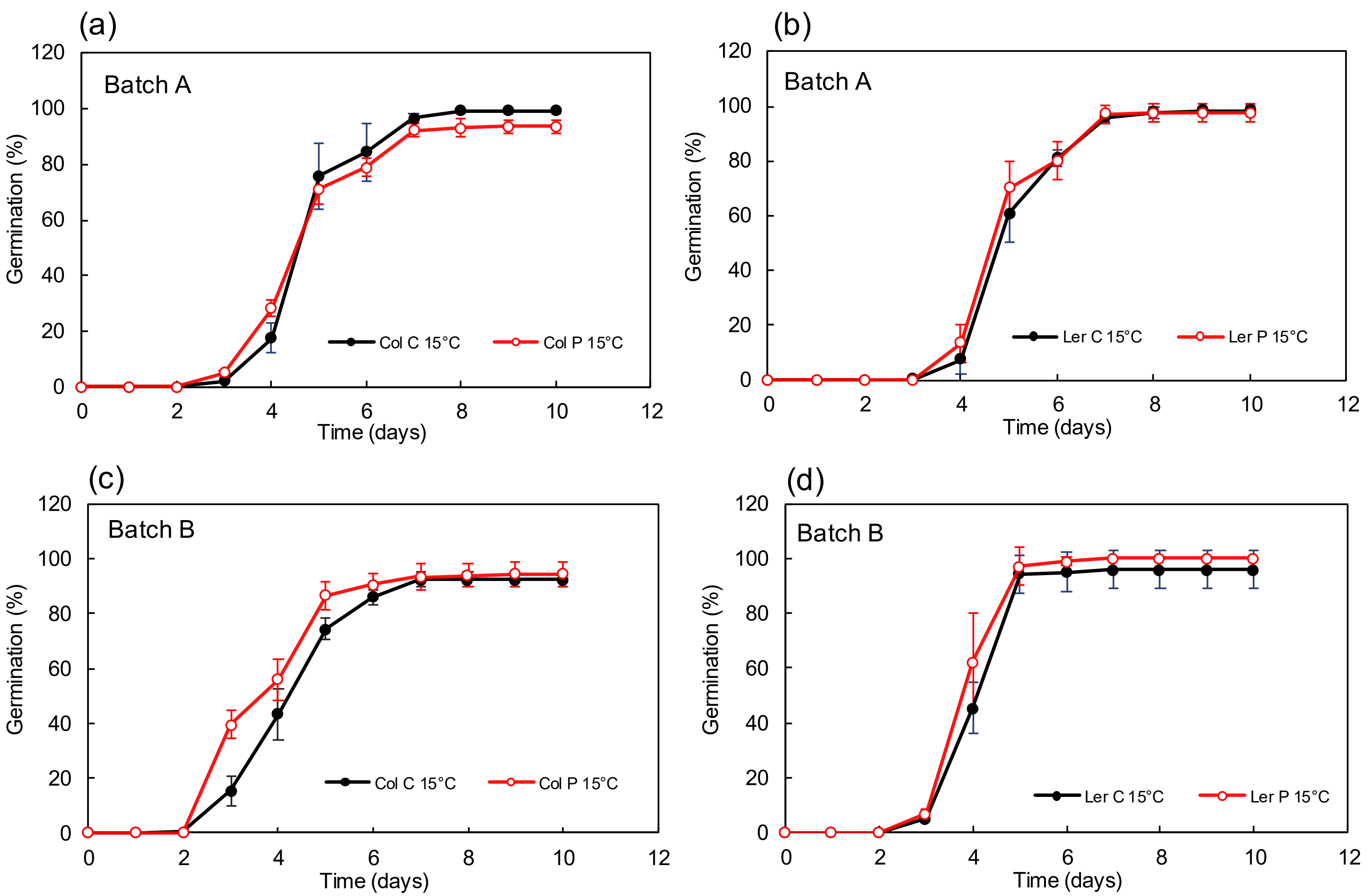

Figure S1: Germination at 15°C of Arabidopsis seeds treated (P) or not (C) by 15 min Cold Atmospheric Plasma. Ecotypes Col (a,c) and Ler (b,c) from 2 batches (A,B) indicated in the figures. Means +/- SD of 3 replicates of 50 seeds.

a)

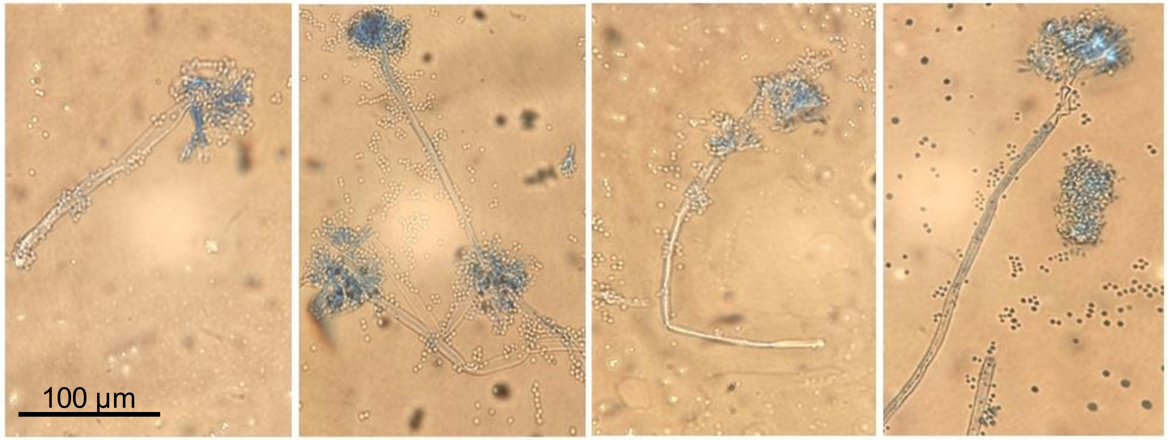

b)

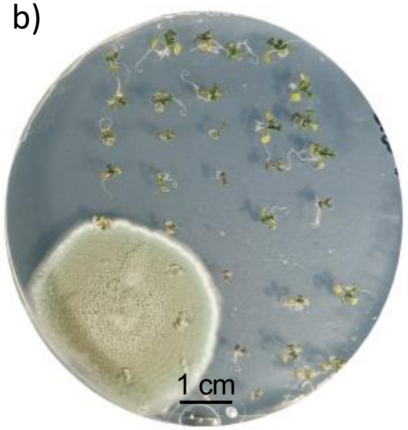

Figure S2: (a) Microscopic observations (×64) of *Penicillium* structures isolated from *Arabidopsis* seeds after lactophenol blue staining, showing characteristic brush-like spore arrangements. b: Colony morphology of *Penicillium* on MS/2 Medium.

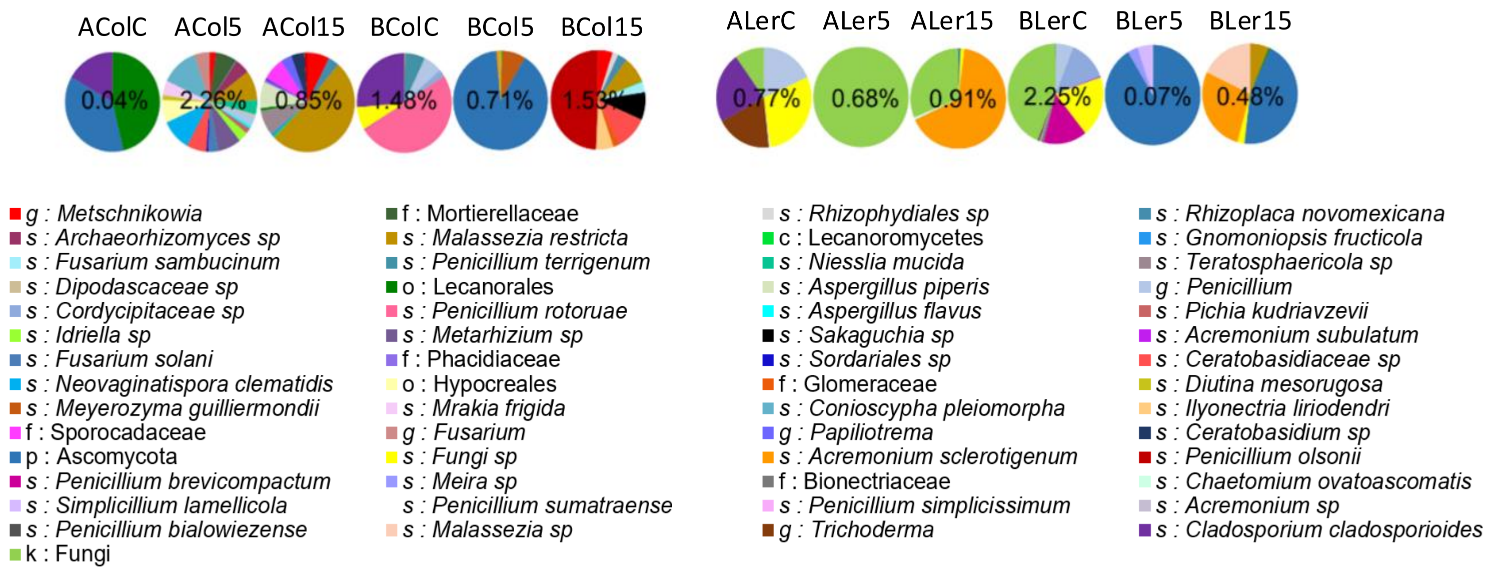

Figure S3: Taxonomic profiles of rare taxa of fungal communities associated with Arabidopsis seedlings of Columbia (Col) and Landsberg (Ler) ecotypes. Fungal diversity associated with seedlings issued from untreated (C) and plasma-treated for 5- or 15- min seeds from two batches (A & B). Each pie chart represents the average relative abundance of rare taxa from 3 samples per condition.

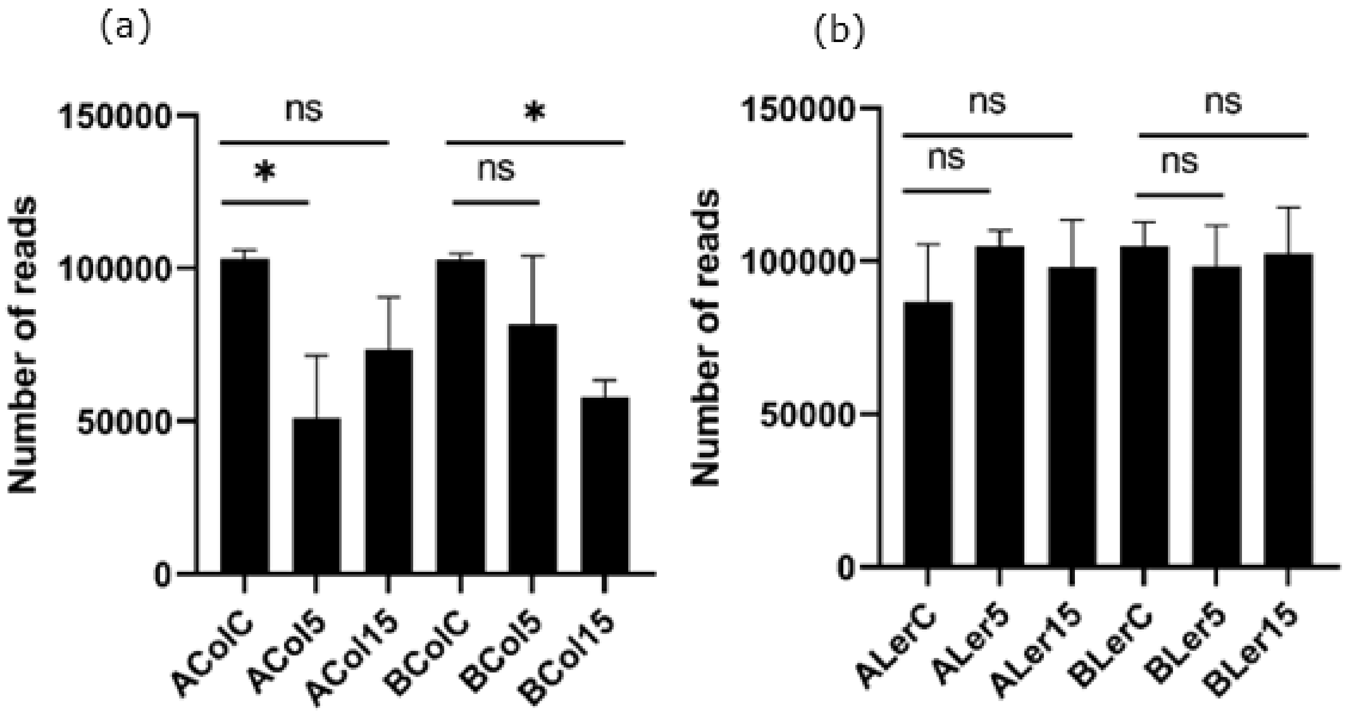

Figure S4: ITS1 region read counts for Columbia (a) and Landsberg (b) in seedlings issued from untreated (C) or plasma-treated for 5- or 15- min seeds from batches A and B. Statistical significance was assessed using an unpaired t-test. Significance levels are indicated as follows: ns ($p > 0.05$), * ($p \leq 0.05$), ** ($p \leq 0.01$), *** ($p \leq 0.001$), **** ($p \leq 0.0001$).
