# Supplementary material for: Cold Atmospheric Plasma Decontaminates Arabidopsis thaliana Seeds and Remodels Seedling Fungal Microbiota
Source: Plants (Basel). 2026 Sep 4;15(17):2719. doi: 10.3390/plants15172719 (PMC13568275; doi:10.3390/plants15172719)
Supplement: Supplementary file 1 [file plants-15-02719-s001.zip › Supplemental tables.pdf]

Table S1 : Alpha diversity indexes for Columbia and Landsberg based on ITS region

Sample	Shannon	Pielou	Chao1	Observed features	Goods coverage
AColC	0.68521836	0.16577108	18.3809524	18.333	0.999
ACol5	2.53814939	0.36365919	117.166667	117	0.999
ACol15	2.38571795	0.34703811	104.733333	104.333	0.999
BColC	1.40402032	0.28718427	29	29	1
BCol5	1.05548656	0.19452278	39.4166667	39.333	0.999
BCol15	3.11323685	0.43121275	150.833333	150.333	0.999
ALerC	0.99296607	0.23988716	17.6666667	17.666	0.999
ALer5	0.25434314	0.06963088	12	12	0.999
ALer15	0.29343045	0.09114556	10.6666667	10.666	0.999
BLerC	1.43811234	0.28656182	32.3333333	32.333	0.999
BLer5	0.89870775	0.23608469	16	16	0.992
BLer15	0.98531905	0.18454257	38.6666667	38.666	0.999

Table S2 : PERMANOVA results for group and batch factors in Columbia and Landsberg.

Ecotype	Factor	Df	Sum of Squares	R^2 (%)	F-value	p-value	Significativity
Columbia	Group	5	3.7942	77.5	7.5741	0.001	***
Columbia	Batch	1	0.2331	4.8	0.7497	0.431	ns
Landsberg	Group	5	2.20493	91.5	25.706	0.001	***
Landsberg	Batch	1	0.47041	19.5	3.879	0.004	**

Table S3 : Pairwise PERMANOVA results for group (A) and time (B) factors in Columbia.

(A)

Group	Pairs		Df	SumsOfSqs	F.Model	R2	p.value	p.adjusted
1	ACol-treated15 vs	ACol-treated5	1	0.006	0.512	0.14	1.0	1.00
2	ACol-treated15 vs	ACol-untreated	1	0.370	39.505	0.90	0.1	0.13
3	ACol-treated15 vs	BCol-treated15	1	0.013	1.566	0.28	0.1	0.13
4	ACol-treated15 vs	BCol-treated5	1	0.272	16.919	0.80	0.1	0.13
5	ACol-treated15 vs	BCol-untreated	1	0.337	34.886	0.89	0.1	0.13
6	ACol-treated5 vs	ACol-untreated	1	0.387	328.121	0.99	0.1	0.13
7	ACol-treated5 vs	BCol-treated15	1	0.002	4.468	0.59	0.1	0.13
8	ACol-treated5 vs	BCol-treated5	1	0.2943	28.911	0.90	0.1	0.13
9	ACol-treated5 vs	BCol-untreated	1	0.356	223.219	0.98	0.1	0.13
10	ACol-untreated vs	BCol-treated15	1	0.551	750.626	0.99	0.1	0.13
11	ACol-untreated vs	BCol-treated5	1	0.009	1.145	0.22	0.3	0.34
12	ACol-untreated vs	BCol-untreated	1	0.002	1.384	0.25	0.2	0.25
13	BCol-treated15 vs	BCol-treated5	1	0.422	56.385	0.93	0.1	0.13
14	BCol-treated15 vs	BCol-untreated	1	0.5068	485.464	0.99	0.1	0.13
15	BCol-treated5 vs	BCol-untreated	1	0.008	1.005	0.20	0.5	0.53

(B)

Time	Pairs	Df	SumsOfSqs	F.Model	R2	p.value	p.adjusted	sig
1	15 vs 5	1	0.2159165	5.191	0.36	0.052	0.052	.
2	15 vs 0	1	0.8749702	151.681	0.93	0.001	0.003	**
3	5 vs 0	1	0.1861974	5.028	0.358	0.026	0.039	*

Table S4: Pairwise PERMANOVA results for group (A) and time (B) factors in Landsberg.

(A)

Group	Pairs		Df	SumsOfSqs	F.Model	R2	p.value	p.adjusted
1	ALer-treated15 vs	ALer-treated5	1	0.0006823338	1.203	0.23	0.5	0.50
2	ALer-treated15 vs	ALer-untreated	1	0.0849479441	110.727	0.96	0.1	0.10
3	ALer-treated15 vs	BLer-treated15	1	0.0160558036	2.868	0.41	0.1	0.107
4	ALer-treated15 vs	BLer-treated5	1	0.6973550840	206.366	0.98	0.1	0.10
5	ALer-treated15 vs	BLer-untreated	1	0.1167406590	45.995	0.91	0.1	0.10
6	ALer-treated5 vs	ALer-untreated	1	0.0877770089	97.036	0.96	0.1	0.10
7	ALer-treated5 vs	BLer-treated15	1	0.0135712315	2.366	0.37	0.1	0.10
8	ALer-treated5 vs	BLer-treated5	1	0.6788136053	193.030	0.97	0.1	0.10
9	ALer-treated5 vs	BLer-untreated	1	0.1184681461	44.278	0.91	0.1	0.10
10	ALer-untreated vs	BLer-treated15	1	0.0582138058	9.809	0.71	0.1	0.10
11	ALer-untreated vs	BLer-treated5	1	0.6624979056	178.234	0.97	0.1	0.10
12	ALer-untreated vs	BLer-untreated	1	0.0136732853	4.754	0.54	0.1	0.10
13	BLer-treated15 vs	BLer-treated5	1	0.5667957709	66.317	0.94	0.1	0.10
14	BLer-treated15 vs	BLer-untreated	1	0.0716811071	9.302	0.69	0.1	0.10
15	BLer-treated5 vs	BLer-untreated	1	0.5383441707	98.096	0.96	0.1	0.10

(B)

Time	Pairs	Df	SumsOfSqs	F.Model	R2	p.value	p.adjusted	sig
1	15 vs 5	1	0.2917675	3.989581	0.28	0.112	0.112	ns
2	15 vs 0	1	0.1509272	23.723086	0.70	0.004	0.012	*
3	5 vs 0	1	0.3573002	4.975931	0.33	0.032	0.048	*

Table S5: IST1-1F primer used in this study

Species type	Amplified region	Primer
Fungi	ITS1-1F	CTTGGTCATTTAGAGGAAGTAA.GCTGCGTTCTTCATCGATGC

Table S6: Summarization of each step of processing for sequencing data of fungal ITS. RawPE (250 bp paired-end raw reads) represents original PE reads after sequencing; Combined represents tags merged from PE reads; Clean represents tags after filtering; Effective represents tags after filtering chimera and can be finally used for subsequent analysis; Base is the number of bases of the Effective Tags; AvgLen represents average length of Effective Tags; Q20 and Q30 are the percentages of bases whose quality value in Effective Tags is greater than 20 (sequencing error rate is less than 1%) and 30 (sequencing error rate is less than 0.1%); GC (%) represents GC content in Effective Tags. Col. ecotype Columbia; Ler. ecotype Lansberg; C. intreated control; 5. 5 min CAP treatment; 15. 15 min CAP treatment. Replicates are indicated as 1. 2 and 3

Sample	RawPE	Combined	Qualified	Nochime	Base(nt)	Avglen(nt)	GC	Q20	Q30
AITSColC1	105743	105111	104087	103067	25580227	248.19	53.14%	99.23%	97.79%
AITSColC2	109893	109368	108476	107807	26934170	249.84	52.98%	99.36%	97.99%
AITSColC3	104819	104235	103477	102981	25693985	249.5	52.98%	99.24%	97.78%
AITSCol51	105276	98934	86507	85664	25197001	294.14	51.59%	93.57%	86.42%
AITSCol52	56785	53341	43443	43072	14785144	343.27	54.75%	91.64%	82.80%
AITSCol53	106347	99999	82761	81968	28331658	345.64	54.97%	91.60%	82.70%
AITSCol151	104220	99195	87333	86742	25811835	297.57	52.73%	93.38%	85.91%
AITSCol152	105839	104183	100360	100018	24934469	249.3	52.67%	97.84%	95.02%
AITSCol153	111386	104151	85400	84584	27372192	323.61	53.72%	92.33%	84.25%
AITSLerC1	67418	67192	67081	66248	16181475	244.26	53.02%	99.56%	98.40%
AITSLerC2	92592	92289	92133	91241	22348582	244.94	53.01%	99.59%	98.45%
AITSLerC3	105349	105032	104782	103396	25303224	244.72	53.01%	99.65%	98.57%
AITSLer51	102765	102359	102056	101766	25283761	248.45	52.90%	99.58%	98.42%
AITSLer52	112642	112141	111622	111293	27692258	248.82	52.88%	99.39%	98.07%
AITSLer53	104659	104162	103614	103164	25574620	247.9	52.86%	99.49%	98.20%
AITSLer151	86392	86014	85724	82311	20450200	248.45	52.88%	99.45%	98.14%
AITSLer152	113865	113426	113064	112772	28012031	248.4	52.87%	99.46%	98.19%
AITSLer153	103045	102658	102395	100011	24794808	247.92	52.87%	99.62%	98.47%
BITSColC1	105853	105464	105256	103827	25394807	244.59	52.85%	99.52%	98.30%
BITSColC2	106354	106009	105786	104635	25605711	244.71	52.87%	99.67%	98.62%
BITSColC3	102343	102019	101766	100851	24841533	246.32	52.83%	99.66%	98.58%
BITSCol51	102328	101391	99492	99147	25312362	255.3	53.01%	98.82%	97.02%
BITSCol52	103362	102133	99107	98766	25576678	258.96	53.12%	98.16%	95.77%
BITSCol53	104130	99537	84058	83419	26070548	312.53	53.88%	93.01%	85.97%
BITSCol151	109278	103468	86538	85778	27422600	319.69	53.72%	92.16%	83.58%
BITSCol152	103809	98266	82743	82107	25310587	308.26	53.02%	92.92%	85.15%
BITSCol153	100193	95904	81340	79986	25753300	321.97	53.27%	92.33%	84.32%
BITSLerC1	103016	102632	102398	101056	24640763	243.83	52.98%	99.66%	98.61%
BITSLerC2	116539	116167	116000	114157	27694508	242.6	53.04%	99.72%	98.77%
BITSLerC3	102311	101962	101765	100295	24453959	243.82	53.00%	99.66%	98.60%

BITSLer51	86198	85734	85359	84768	20384856	240.48	52.13%	99.36%	97.90%
BITSLer52	112693	112043	111799	111505	25968449	232.89	52.03%	99.46%	97.99%
BITSLer53	106804	106025	104751	104656	24848541	237.43	51.95%	98.93%	97.02%
BITSLer151	155168	151611	140705	139995	39388239	281.35	53.02%	96.09%	91.75%
BITSLer152	102368	101022	97908	97648	25239193	258.47	53.14%	98.33%	96.02%
BITSLer153	106543	105060	101804	101456	26300258	259.23	53.14%	98.16%	95.71%
